# Genome-Wide Identification and Analysis of MAPK and MAPKK Gene Families in *Brachypodium distachyon*

**DOI:** 10.1371/journal.pone.0046744

**Published:** 2012-10-17

**Authors:** Lihong Chen, Wei Hu, Shenglong Tan, Min Wang, Zhanbing Ma, Shiyi Zhou, Xiaomin Deng, Yang Zhang, Chao Huang, Guangxiao Yang, Guangyuan He

**Affiliations:** 1 The Genetic Engineering International Cooperation Base of Chinese Ministry of Science and Technology, Chinese National Center of Plant Gene Research (Wuhan) HUST Part, College of Life Science and Technology, Huazhong University of Science & Technology (HUST), Wuhan, China; 2 Services Computing Technology and System Laboratory, Cluster and Grid Computing Laboratory, School of Computer Science and Technology, Huazhong University of Science & Technology (HUST), Wuhan, China; National Taiwan University, Taiwan

## Abstract

MAPK cascades are universal signal transduction modules and play important roles in plant growth, development and in response to a variety of biotic and abiotic stresses. Although MAPKs and MAPKKs have been systematically investigated in several plant species including *Arabidopsis*, rice and poplar, no systematic analysis has been conducted in the emerging monocot model plant *Brachypodium distachyon*. In the present study, a total of 16 MAPK genes and 12 MAPKK genes were identified from *B. distachyon*. An analysis of the genomic evolution showed that both tandem and segment duplications contributed significantly to the expansion of MAPK and MAPKK families. Evolutionary relationships within subfamilies were supported by exon-intron organizations and the architectures of conserved protein motifs. Synteny analysis between *B. distachyon* and the other two plant species of rice and *Arabidopsis* showed that only one homolog of *B. distachyon* MAPKs was found in the corresponding syntenic blocks of *Arabidopsis*, while 13 homologs of *B. distachyon* MAPKs and MAPKKs were found in that of rice, which was consistent with the speciation process of the three species. In addition, several interactive protein pairs between the two families in *B. distachyon* were found through yeast two hybrid assay, whereas their orthologs of a pair in *Arabidopsis* and other plant species were not found to interact with each other. Finally, expression studies of closely related family members among *B. distachyon*, *Arabidopsis* and rice showed that even recently duplicated representatives may fulfill different functions and be involved in different signal pathways. Taken together, our data would provide a foundation for evolutionary and functional characterization of MAPK and MAPKK gene families in *B. distachyon* and other plant species to unravel their biological roles.

## Introduction

The mitogen activated protein kinases (MAPK) pathway is conserved in evolution through the plant and animal kingdoms. These proteins have been implicated in diverse cellular processes including cell growth, proliferation, differentiation, survival, development and in responses to a diversity of environmental stimuli including cold, heat, reactive oxygen species, UV, drought and pathogen attack [1]–[4]. MAPK cascades are composed of three classes of protein kinases: MAPK kinase kinase (MAPKKKs/MEKKs), MAPK kinase (MAPKKs/MKKs) and MAPK. MAPKKKs are serine/threonine kinases phosphorylating two amino acids in the S/T-X3–5-S/T motif of the MAPKK activation loop. MAPKKs are dual-specificity kinases that activate a MAPK through double phosphorylation of the T-X-Y motif in the activation loop (T-loop). The activated MAPK leads to the phosphorylation of transcription factors and other signaling components that regulate the expression of downstream genes [5].

To date, there have been only a few MAPK cascade components that have been studied in detail in plants [4] and the most extensively studied MAPKs are MPK3, MPK4 and MPK6 in *Arabidopsis* and crop plants [5]. The *Arabidopsis* MKK1/MKK2-MPK4/MPK6 cascades have previously been shown to play important roles in the responses to salt and cold stresses and pathogen attack [6]–[9]. The *Arabidopsis* MKK3 participates in signaling cascades elicited by pathogen infection [10]. The *Arabidopsis* MKK4/MKK5-MPK3/MPK6 cascades play important roles in the regulation of biotic stress [11]–[12]. The *Arabidopsis* MPK4 and the rice OsMAPK3 negatively regulate biotic stress signaling [4], [13]. In addition, several MAPK genes have been found to play an important role in the regulation of developmental processes [11], [14]–[16]. A tobacco MAP Kinase p45^ntf4^ was identified and proven to be involved in pollen maturation and germination and be activated by hydration during pollen germination [17]. The *Arabidopsis* MKK1-MPK3/MPK6 cascades are key players in stomatal development and stomatal dynamics [12], [18]–[19]. The *Arabidopsis* MKK9-MPK3/MPK6 cascades regulate ethylene signaling and camalexin biosynthesis and may also play a role in leaf senescence [20]–[22]. The *Arabidopsis* MKK6-MPK4/MPK11 cascades directly regulate cytokinesis and mitosis [23]–[26].

So far, a large number of members of MAPK cascades from *Arabidopsis* and rice have been identified using functional genomic approach. Twenty MAPKs, 10 MAPKK and 80 MAPKKKs are present in the *Arabidopsis thaliana* genome and 15 MAPKs, 13 MAPKK and 75 MAPKKKs are present in rice [27]–[28]. However, no systematic investigations of MAPK and MAPKK families have been reported in *B. distachyon*. The *B. distachyon* is fast emerging as a powerful model system to study the functional genomics of the temperate grasses, cereals, and dedicated biofuel crops such as Switchgrass [29]. The attributes include small genome (∼300 Mb) diploid accessions, a small physical stature, self-fertility, a short lifecycle and simple growth requirements. Recently, the *B. distachyon* genome (diploid inbred line Bd21) has been sequenced [30], which provides an unprecedented opportunity for systematic analysis of the MAPK cascades in the *B. distachyon* species.

In the present study, 16 MAPK genes and 12 MAPKK genes were identified from the Bd21 genome by database searches and they were classified according to their homology with known MAPK and MAPKK genes in *Arabidopsis* or rice. We evaluated their expansion and evolutionary mechanisms by their duplication, distribution on the chromosomes and synteny map with their orthologs in *Arabidopsis* and rice and examined their protein interaction profile by yeast two-hybrid (Y2H) assay between these two families. Subsequently, we investigated their transcript profilings in different organs and in response to different stresses by RT-PCR and that of their orthologs in *Arabidopsis* and rice using Affymetrix *Arabidopsis* and rice microarray data. Finally, we conducted the pairwise comparisons of the expression profiles of putative orthologous or paralogs pairs existing in the two family genes of *B. distachyon* and rice under different light and temperature conditions. Our results from *B. distachyon* would provide the basis for future research on their expansion and evolutionary mechanisms and the diverse signaling pathways medicated by MAPKs and MAPKKs in *B. distachyon* and are more likely to be directly translatable to agriculturally important monocots, grasses such as wheat and barley by transferring functional information between species [31].

## Materials and Methods

### Identification, sequence alignment and phylogenetic analyses of MAPK and MAPKK genes in *B. distachyon*

The *B. distachyon* genome assembly version 1.2 was downloaded from the website ftp://ftp.brachypodium.org/to construct a local protein database. Method used to identify the MAPK and MAPKK genes in *B. distachyon* was similar to that described in rice and poplar [19]. For the MAPKK gene family, the predicted proteins derived from *B. distachyon* pseudo-molecules were queried using a profile Hidden Markov Model-based search (HMMER: http://hmmer.wustl.edu/) with an HMM built from the ten *Arabidopsis* MAPKKs [32]. MAPKK gene models were only accepted if they displayed the consensus sequences for dual-specificity protein kinases, including the conserved aspartate and lysine residues within the active site motif (–D(L/I/V)K-), and the plant-specific phosphorylation target site motif (–S/TxxxxxS/T-) within the activation loop. Similarly, the predicted *B. distachyon* proteins were queried using a profile Hidden Markov Model-based search with an HMM built from the twenty *Arabidopsis* MAPKs for the MAPK gene family. MAPK gene models were only accepted if they contained the canonical consensus sequences for serine/threonine protein kinases, as well as an appropriately positioned activation loop (-TXY-motif). And then the predictions of MAPK and MAPKK coding sequences were verified with available EST. Finally, multiple alignments of the identified *B. distachyon* amino acid sequences of these two gene families with that of *Arabidopsis* and rice were performed by Clustal W [33] with default options and the phylogenetic trees were constructed based on the bootstrap neighbor-joining (NJ) method with a Kimura two-parameter model by MEGA [34].

### Analyses of the conserved motifs, chromosomal locations, gene duplication, gene structures, promoter regions and synteny with the homologs of rice and *Arabidopsis*

We performed the MEME (Multiple Expectation maximization for Motif Elicitation) analysis on the predicted candidate proteins of MAPK and MAPKK gene families with the conditions: 1. optimum motif width was set to 6 and 50; 2. maximum number of motifs was designed to identify 10 motifs; 3. the iterative cycles were set by default [35]. The exon/intron structures and locations for the two gene families were confirmed by Blat (http://genome.ucsc.edu/FAQ/FAQblat.html) search using a local database containing the complete *B. distachyon* genome sequences of each chromosome. Moreover, the gene duplication events of *B. distachyon* MAPK and MAPKK genes were also investigated. We defined the gene duplication in accordance with the criteria: 1. the alignment length covered ≥70% of the longer gene; 2. the aligned region had an identity ≥70%; 3. only one duplication event was counted for tightly linked genes [36]. A block of duplications was defined if more than one gene was involved in the duplication.

To analyze their promoter regions, the 1 kb upstream regions of the two gene families according to the position of the genes provided by the *B. distachyon* annotation information were selected and screened against the PLACE database [37]. Regulatory elements overrepresented in the dataset and known to be involved in regulation during the resistance response and under stressed conditions were selected for further analysis [38]. Among them, WBOX [sequence TGAC(C/T)] associated with the WRKY transcription factors [39], CBF (GTCGAC), DRE [(G/A)CCGAC] [40] and GCC boxes associated with the ERF-type transcription factors [41] were retained for further analysis. For synteny analysis, synteny blocks between *B. distachyon* and the other two plant species of rice and *Arabidopsis* were downloaded from the Plant Genome Duplication Database [42] and those containing *B. distachyon* MAPK and MAPK genes were identified, respectively.

### cDNA cloning, plasmid constructions and yeast two-hybrid assays

Six MAPK and four MAPKK genes with more EST counts support, respectively, were selected to be amplified by PCR using primers designed based on the predicted sequences (listed in Supplemental Table S1). A complete list of primers used for RT-PCR, cDNA cloning and expression studies during the course of this work were provided in supplemental Table S1. Plasmid constructions and yeast two-hybrid assay for the two family genes were performed as described by Albrecht et al. [43]. All the yeast two-hybrid assays were performed in triplicate.

### Plant materials, growth conditions, stress treatments and expression profile analyses of the two family genes in *B. distachyon* by RT-PCR

The community standard diploid inbred line of *B. distachyon*, Bd21, was used for all the experiments. After germination, they were planted in greenhouse and were grown under natural light and temperature conditions. The 3-week-old seedlings were used for all treatments. For drought, salinity, cold, abscisic acid (ABA), H_2_O_2_ and methyl jasmonate (MeJA) treatments, seedlings were subjected to 20% polyethylene glycol (PEG), 200 mM NaCl, 4°C conditions, 100 µM ABA, 10 mM H_2_O_2_ and 100 µM MeJA, respectively. The leaves of all the samples for RNA extractions were collected at 3 h after treatments. The roots, stems, leaves and caryopses of more than two-month old Bd21 were collected separately for RNA isolation and used for organ-specific expression analysis.

Total RNA was isolated according to the method described in the grape [44]. For RT-PCR, the specific primers were designed according to the predicted two family gene sequences by Primer 5 software (Table S1). A *B. distachyon* β-actin gene (ID: Bradi2g24070.1), amplified with primers 5′- CCCGATGGACAGGTTATCACTA-3′ and 5′- ATAGAGCCACCAATCCAAACAC-3′, was used as a control. The following program was used for RT-PCR: 94°C for 5 min followed by 35 cycles at 94°C for 10 s, 55–59°C for 15 s and 72°C for 30 s, followed by a 5 min extension step at 72°C. While the number of cycles of PCR for actin gene was set as 30. The experiments were repeated at least three times with independent RNA samples.

### Expression analyses of the two family genes in *Arabidopsis* and rice

Affymetrix *Arabidopsis* and rice microarray data were downloaded from ArrayExpress [45], PLEXdb [46] and Rice Oligonucleotide Databases [47]. Expression intensities in different development stages are log 2-based values and then were used for the tissue expression analysis. For the abiotic stress treatments, expression patterns of all samples (at least two biological repeats) were transformed into log 2-based numbers and normalized according to the quantile method for standardization of array data. Expression of a gene (up-or down-regulated) was defined as a gene with a log 2-based ratio (Cold, drought and salt-stressed sample/mock sample) higher than 0.5 or lower than −0.5; and a significant difference in gene expression between the treated plants and the control indicated by ≤0.05 by paired t-test [permutations, all possible combinations; false discovery rate (FDR) correction, adjusted Bonferroni method] [48]. Hierarchical clustering of expression profiles of the two family genes in *Arabidopsis* and rice was performed using R version 2.15.1.

### Pairwise comparisons of the expression profiles of putative orthologous or paralogs existing in the two family genes of *B. distachyon* and rice under different light and temperature conditions

Available expression data of the two family genes of *B. distachyon* and rice under different light and temperature conditions were downloaded from PLEXdb [46] and Rice Oligonucleotide Databases [47]. All expression data (relative amount of mRNA) were composed of thirty nine treatment points. The Pearson correlation of the expression of orthologs or paralogs pairs existing in the two family genes was calculated on the basis of the expression data under the corresponding light and temperature conditions.

## Results and Discussion

### Identification and annotation of the *B. distachyon* MAPK and MAPKK families

Availability of complete *B. distachyon* genome sequences has made it possible for the first time to identify all the MAPKK and MAPK family members in this plant species. In order to determine the two family genes, we performed HMM searches and identified 16 MAPK and 12 MAPKK genes, respectively, from *B. distachyon* genomes. Based on *Arabidopsis* MAPK and MAPKK nomenclature suggestions [27], each gene was named with a two-letter code corresponding to *B. distachyon* (*Bd*), followed the family designation (*MPK* or *MKK*), and finally a number (Table 1). Because there was the occurrence of alternative mRNA splicing in some genes of the two families, the following analysis was restricted to the longest protein for each gene except specific description.

**Table 1:** Nomenclature for MAPKs and MAPKKs in *Arabidopsis*, *Brachypodium* and *Oryza.*

Family	Bd gene name	Bd gene model	At gene name*	At gene code*	Os gene name*	Os gene code*
**MAPK**	*BdMPK3*	Bradi1g65810	*AtMPK1*	At1g10210	*OsMPK3*	Os03g17700
	*BdMPK4*	Bradi3g32000	*AtMPK2*	At1g59580	*OsMPK4*	Os10g38950
	*BdMPK6*	Bradi1g49100	*AtMPK3*	At3g45640	*OsMPK6*	Os06g06090
	*BdMPK7-1*	Bradi1g34030	*AtMPK4*	At4g01370	*OsMPK7*	Os06g48590
	*BdMPK7-2*	Bradi4g24912	*AtMPK5*	At4g11330	*OsMPK14*	Os02g05480
	*BdMPK11*	Bradi3g16560	*AtMPK6*	At2g43790	*OsMPK16*	Os11g17080
	*BdMPK14*	Bradi3g03780	*AtMPK7*	At2g18170	*OsMPK17-1*	Os06g49430
	*BdMPK16*	Bradi2g36470	*AtMPK8*	At1g18150	*OsMPK17-2*	Os02g04230
	*BdMPK17*	Bradi1g34700	*AtMPK9*	At3g18040	*OsMPK20-1*	Os01g43910
	*BdMPK20-1*	Bradi2g44350	*AtMPK10*	At3g59790	*OsMPK20-2*	Os05g50560
	*BdMPK20-2*	Bradi2g15317	*AtMPK11*	At1g01560	*OsMPK20-3*	Os06g26340
	*BdMPK20-3*	Bradi1g41780	*AtMPK12*	At2g46070	*OsMPK20-4*	Os01g47530
	*BdMPK20-4*	Bradi2g45870	*AtMPK13*	At1g07880	*OsMPK20-5*	Os05g49140
	*BdMPK20-5*	Bradi2g16337	*AtMPK14*	At4g36450	*OsMPK21-1*	Os05g50120
	*BdMPK21-1*	Bradi2g15620	*AtMPK15*	At1g73670	*OsMPK21-2*	Os01g45620
	*BdMPK21-2*	Bradi2g45010	*AtMPK16*	At5g19010		
			*AtMPK17*	At2g01450		
			*AtMPK18*	At1g53510		
			*AtMPK19*	At3g14720		
			*AtMPK20*	At2g42880		
**MAPKK**	*BdMKK1*	Bradi1g51000	*AtMKK1*	At4g26070	*OsMKK1*	Os06g05520
	*BdMKK3-1*	Bradi4g39490	*AtMKK2*	At4g29810	*OsMKK3*	Os06g27890
	*BdMKK3-2*	Bradi1g41860	*AtMKK3*	At5g40440	*OsMKK4*	Os02g54600
	*BdMKK3-3*	Bradi3g11260	*AtMKK4*	At1g51660	*OsMKK5*	Os06g09180
	*BdMKK4*	Bradi3g53650	*AtMKK5*	At3g21220	*OsMKK6*	Os01g32660
	*BdMKK5*	Bradi1g46880	*AtMKK6*	At5g56580	*OsMKK10-1*	Os02g46760
	*BdMKK6*	Bradi1g75150	*AtMKK7*	At1g18350	*OsMKK10-2*	Os03g12390
	*BdMKK10-1*	Bradi1g11525	*AtMKK8*	At3g06230	*OsMKK10-3*	Os03g50550
	*BdMKK10-2*	Bradi1g69400	*AtMKK9*	At1g73500		
	*BdMKK10-3*	Bradi1g10800	*AtMKK10*	At1g32320		
	*BdMKK10-4*	Bradi1g10770				
	*BdMKK10-5*	Bradi1g10790				

* From Hamel et al [19]. At: *Arabidopsis thaliana*, Os: *Oryza sativa*, Bd: *B.distachyon*.

To assess which gene in this study had expression support, we compared the predicted genes against available ESTs (http://www.brachypodium.org/). Because many members of these two families are similar to one another, only top matches were considered, after applying a high match stringency of at least 95% of nucleotide identity between EST and genomic sequences (Table S2). At this threshold, 7 MAPKK genes and 14 MAPKK genes in this study had EST supports, representing 87.5% and 58.3% of predicted MAPKK and MAPK genes in the study, respectively. Four MAPKK genes (six proteins generated by alternative splicing) and six MAPK genes with more EST counts support were selected to be amplified by PCR for the following protein interactive analysis. All the cDNAs of our cloned genes of the two families have been submitted to GenBank and their accession numbers in GenBank were shown in Table S2.

### Multiple alignments, phylogenetic and domain analyses of MAPK and MAPKK families

**Figure 1:**
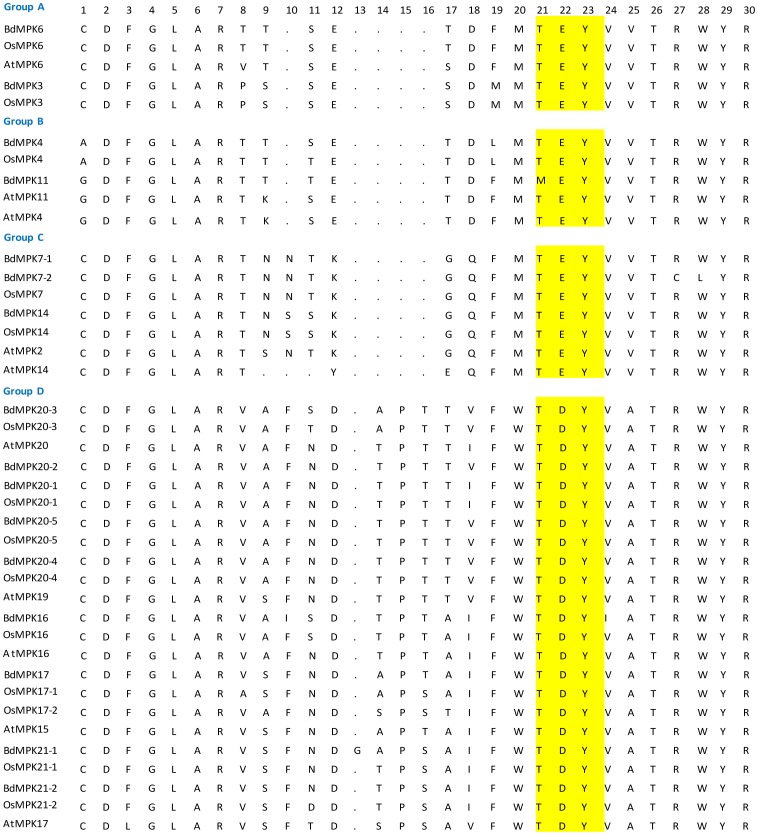
Alignment of multiple *B. distachyon* MAPK, selected *Arabidopsis* and rice MAPK domain amino acid sequences. Alignment was performed using Clustal W. The conserved T-loop amino acid signature is highlighted in yellow, and gaps are marked with dot lines.

**Figure 2:**
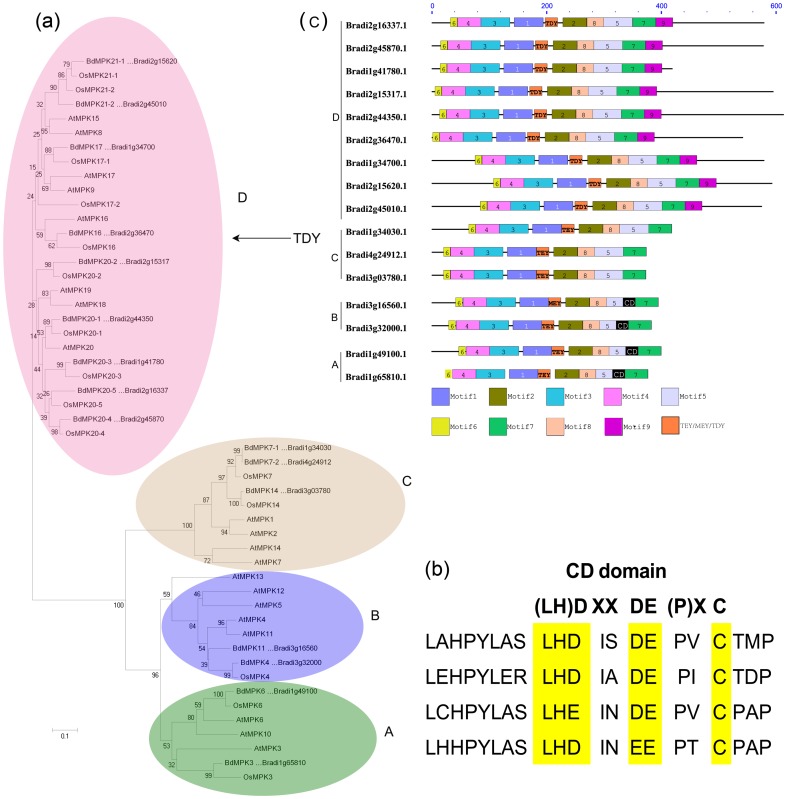
Phylogenetic and domain analyses of MAPKs. (a) Phylogenetic relationships of *B. distachyon*, *Arabidopsis* and rice MAPK genes. The phylogenetic tree was constructed by NJ (Neighbor-joining) method using the MEGA 4 program. Different color patternings indicate different gene clusters or superclusters. (b) Comparison of CD domain among four BdMAPKs (c) Schematic diagram of amino acid motifs of *B. distachyon* MAPKs from different groups. Motif analysis was performed using MEME 4.0 software as described in the methods. The black solid line represents the corresponding BdMAPK and its length. The different-colored boxes represent different motifs and their position in each BdMAPK sequence. A detailed motif introduction for all the *B. distachyon* MAPKs is shown in Figure S1.

The phylogenetic relationship of the MAPK family proteins in *B. distachyon* was examined by multiple sequence alignment of their conserved protein kinase, which spans approx. about 300 amino acids. The alignment analysis based on T-loop motif sequences of MAPK family from thee species divided them into four groups (Figure 1). As shown in Figure 1, the sequences in the T-loop motif were highly conserved. A comparison with the protein kinase motifs of the MAPK representative proteins from *Arabidopsis* and rice could result in a better separation of the different groups and subgroups. Through phylogenetic analyses of the conserved motifs of the three species, we found that the BdMAPKs could also be classified into four groups corresponding to the groups A, B, C and D in *Arabidopsis* (Figure 2a) [49]. Those *B. distachyon* and rice MAPKs that harbored a -TEY- signature in their activation loop clustered with their *Arabidopsis* homologs except BdMAPK11 in which the Met-Glu-Tyr (-MEY-) replaced the -TEY- signature; while the large clade of group D MAPKs all displayed a distinctive -TDY- rather than -TEY- signature (Figure 1 and Figure 2a). This case that “MEY” replaced the “TEY” signature was also observed in tomato but not observed in rice [50], even though the relationship of the two monocots species *B. distachyon* and rice are evolutionarily more closely than that of *B. distachyon* and dicots tomato.

Moreover, four MAPK genes (*BdMPK3, BdMPK4, BdMPK6 and BdMPK11*) belonging to group A and B were found to have a CD domain in their C-terminal region, which functioned as a binding site of MAPKKs and conserved as a DxxDE(P)xC motif in the evolution from the multiple sequence alignment results (Figure 2b). Group C and D did not possess such a CD domain, but group D had a relatively long C-terminal region. The results were consistent with that of MEME results (Figure 2c). A total of ten motifs were identified from the BdMPK proteins. Nine of ten motifs corresponded with the eleven domains (I–XI) that are found in *B. distachyon* serine/threonine protein kinases (Figure S1). From the MEME results, we could also find that all the members of large group D contained the extra motif 9 besides their common motifs shared with other subgroup A, B and C (Figure 2c). The last subdomain 11 of serine/threonine protein kinases was corresponding with the motif 7, while no subdomains corresponded with the motif 9, which only existed in the group D and followed behind the motif 7. Whether the difference between the large group D and other subgroups A, B and C might have some effect on their biological function needs to be further studied in the future. However, so far relatively little work has been done to identify the function of MAPKs in the large group D.

**Figure 3:**
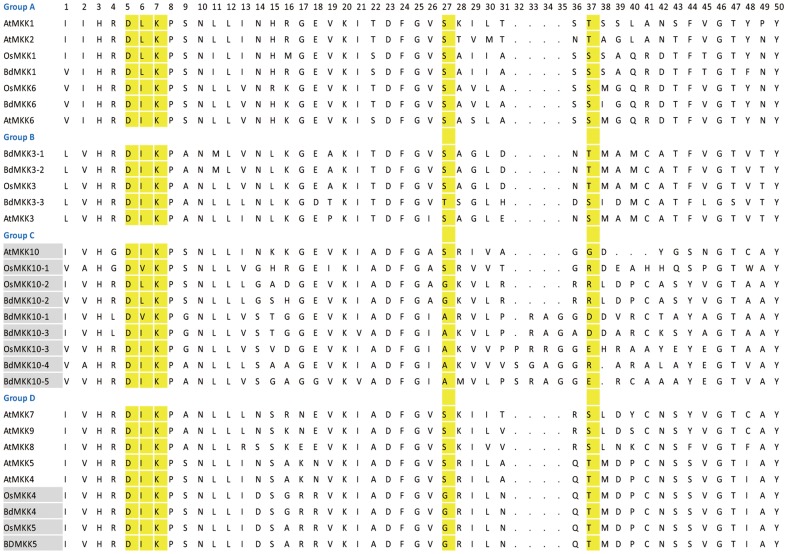
Alignment of multiple *B. distachyon* MAPKK, selected *Arabidopsis* and rice MAPKK domain amino acid sequences. Alignment was performed using Clustal W. The conserved S/T-X5-S/T motif and active site D(I/L/V)K motif were highlighted in yellow. The genes with incomplete activation loop motif in which the 5′- or 3′- S/T residue is either absent or located 3–5 residues downstream of the canonical position were highlighted in grey. And gaps are marked with dot lines.

For the *B. distachyon* MAPKKs, similar work was done as the MAPK gene family. Animal MAPKKs had the S/T-X5-S/T motif as the phosphorylation site which was also observed in some fungi and many plant MAPKKK kinases (MEKKKs). Comparison analysis between the amino acid sequences of *B. distachyon*, *Arabidopsis* and rice MAPKK S/T-X5-S/T motifs was shown in Figure 3. The results revealed that the activation loop motif of four members of the ‘Group D’ (*OsMKK4*, *OsMKK5*, *BdMKK4* and *BdMKK5*) and all the members of ‘Group C’ was either absent or located 3–5 residues downstream of the canonical position (Figure 3), especially the *BdMKK10-1*, *BdMKK10-3*, *BdMKK10-4*, *BdMKK10-5* and *OsMKK10-3* which were more divergent in the S/T-X5-S/T motif compared with others. More interestingly, all these four genes had no EST support and the expression profiles of their orthologous in *Arabidopsis* and rice were also not detected by RT-PCR in the two species [19]. In order to find the convincing evidence to determine whether these genes with untypical domain were expressed in a certain condition, they were subjected to RT-PCR and microarray data analysis in the following section.

**Figure 4:**
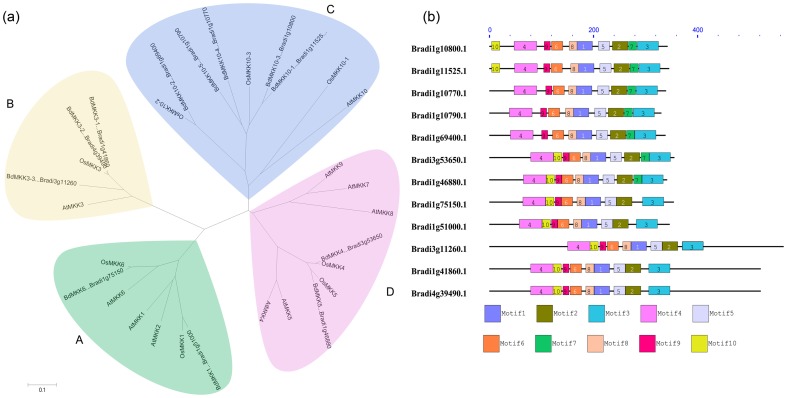
Phylogenetic and domain analyses of MAPKKs. (a) Phylogenetic relationships of *B. distachyon*, *Arabidopsis* and rice MAPKK genes. The phylogenetic tree was constructed by NJ (Neighbor–joining) method using the MEGA 4 program. Different color patternings indicate different gene clusters or superclusters. (b) Schematic diagram of amino acid motifs of *B. distachyon* MAPKKs. The black solid line represents the corresponding BdMAPKK and its length. The different-colored boxes represent different motifs and their position in each BdMAPKK sequence.

Previous study showed that MAPKK proteins from the same subgroup or clade tend to cluster together. However, the evolutionary analysis of MAPKK genes in the three species could not be clarified for all clades and revealed some interesting observations (Figure 4a). For example, most MAPKK genes were placed in generally well-resolved clades that each contained the members from both monocots and eudicots; while the MKK7-9 clade was not the case, for which no *B. distachyon* and rice orthologs cloud be identified for the AtMKK7-9. The different evolutionary patterns of MKK7-9 clade in *Arabidopsis, B. distachyon* and rice might occur after their divergence. Compared with MKK10 clade from *Arabidopsis* (1) and rice (3), *B. distachyon* MKK10 clade was the most expanded with five genes. And MKK10 clade also had the most members in the three species compared with other clades. More interestingly, the relative evolution distance between BdMKK10-2 and other members of its same BdMKK10 clade, was much farther than that between each other of BdMKK10-1, BdMKK10-3 and BdMKK10-4 in the phylogenetic tree, which may be due to the reason that BdMKK10-2 evolved prior to other members in the evolutionary timescale of life.

The conserved motifs of MAPKK family proteins in *B. distachyon* were also investigated using MEME as that of MAPK family, and a schematic overview of the identified motifs was shown in Figure 4b. In the BdMKK10 clade, except that the closely related paralogs BdMKK10-1 and BdMKK10-3 had an extra motif 10 in the N-terminal of their sequence, all other members shared nine conserved motifs with the same organization. However, the motif 10 of other clades was not in the N-terminal of the sequence and all the members of them did not contain the motif 7 except BdMKK4 and BdMKK5 compared with that of BdMKK10 clade. In addition, we also found that all the members of BdMKK3 clade had long terminal sequences both at the 5′ and 3′ terminal besides sharing the conserved motifs with the same organization (Figure 4b). And the genes from *B. distachyon* tend to be clustered more closely with that from rice than that from *Arabidopsis* by comparison with the phylogenic analysis of the two family genes from different species, which may be the reason that *Arabidopsis* and Poaceae split far before the separation of *B. distachyon* and rice.

### Genomic distribution and gene duplication

**Figure 5:**
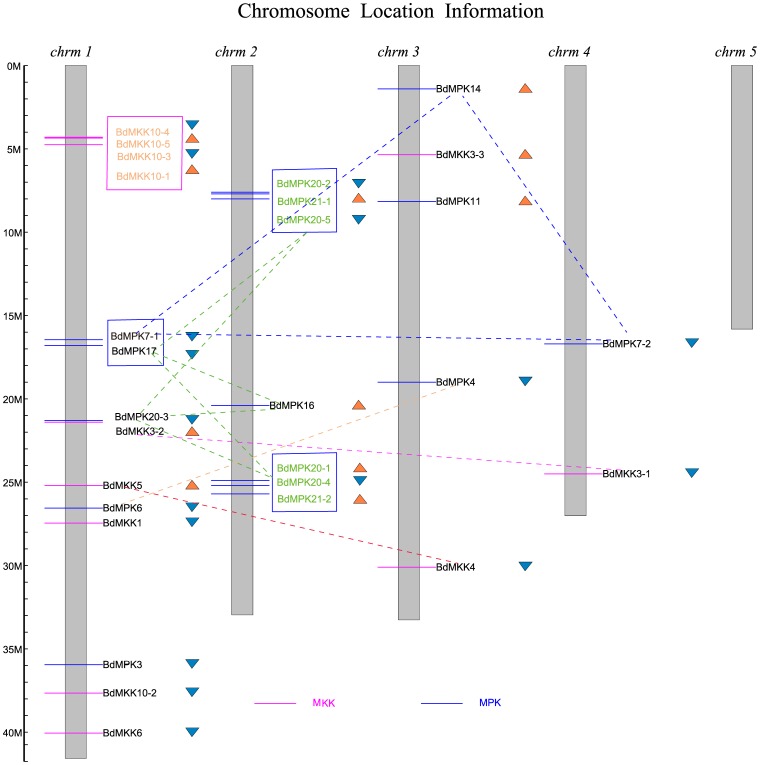
Chromosomal locations of *B. distachyon* MAPK (in blue) and MAPKK (in purple) genes. The MAPK and MAPKK gene clusters were separately indicated with blue and purple rectangles. Different color dotted lines connect the MAPK and MAPKK genes present on duplicate chromosomal segments. The names of each tandem duplicated gene cluster of the two families were indicated with the same color (except the names in black). The orange and blue triangles indicate the upward and downward direction of transcription, respectively.

Figure 5 showed the locations of the MAPK and MAPKK genes on the 5 chromosomes of *B. distachyon*. They were separately located on each chromosome individually or in clusters, and their distribution was non-random. Their chromosomal distribution pattern revealed that certain chromosomes and chromosomal regions had a relatively high density of MAPK or MAPKK genes. For example, none of these two gene family members was located on chromosome 5. Seventy five percent of MAPKK genes were located on the chromosome 1 and the remaining members were respectively located on chromosome 3 and 4. For the MAPK family, the majority of members were respectively located on the chromosome 1, 2 and 3 except BdMPK7-2. Based on Holub's definition of a gene cluster [51], it is a region that contains four or more genes within 200 kb or less. In this study, we found only a gene cluster with four members in the MAPKK family using this criteria and no gene cluster was found in the MAPK family (Figure 5). However, if we relaxed the clustering criteria with at least two genes in a cluster, 69% of MAPK members occurred in clusters but it did not have some effect on the number of MAPKK gene clusters (Figure 5).

We further determined the tandem and segmental duplication events of these two gene families on the five *B. distachyon* chromosomes. As shown in Figure 5, two MAPK gene clusters (The names of each gene cluster was labeled in the same color except the black color) containing 6 tandem duplicated genes were both located on chromosome 2 and one MAPKK gene cluster with four members were located on chromosome 1. Intriguingly, the two gene clusters of MAPK family as well as *BdMPK16, BdMPK17* and *BdMPK 20-3* accounting for 56.25% of MAPK genes were also involved in the segmental duplication event and formed a supercluster, which showed that these members may be originated from chromosomal segment duplication and subsequent divergence under the selective pressure of external environment. The duplicate chromosomal segments of these two families were connected with different color dotted lines. Although several paralogs such as *BdMPK3* and *BdMPK6*, *BdMPK4* and *BdMPK11* shared high similarity of amino acid sequences, they were far from each other on the chromosome and therefore were not considered as tandem duplicated genes. More interestingly, *BdMPK7-1* and *BdMPK 17* were tightly linked on chromosome 1, but they only shared 44.35% similarity of amino acids sequences and were also not considered as tandem duplicated genes, which may be evolved from different progenitor genes. The result was consistent with that of phylogenetic and domain analysis mentioned above and the two genes were located on different subgroups of the MAPK phylogenetic tree. *BdMPK7-1* and *BdMPK 17* were in the subgroup C and D, respectively (Figure 2). However, *BdMPK7-1*, *BdMPK7-2* and *BdMPK14* located on different chromosomes had higher similarity in their amino acids sequence than that of other paralogs, reached up to more than 90% similarity between each other and happened to cluster together in the phylogenic tree, which showed that these genes were possibly originated from recent inter-chromosomal gene duplication event. In addition, we also found that although the *BdMKK10-2* and the gene cluster consisting of other members of *BdMKK10* subgroup were all located on the same chromosome, they were far from each other in the physical distance, which was consistent with the result of MAPKK phylogenetic and multiple alignment analysis. Taken together, a total of 87.5% of MAPK genes and 66.7% of MAPKK genes were the results of either tandem duplication or segmental duplication event, which suggested that the two duplication patterns played a major role in the expansion of the two families in *B. distachyon*.

### Analyses of the MAPK and MAPKK gene structures and their promoter regions

**Figure 6:**
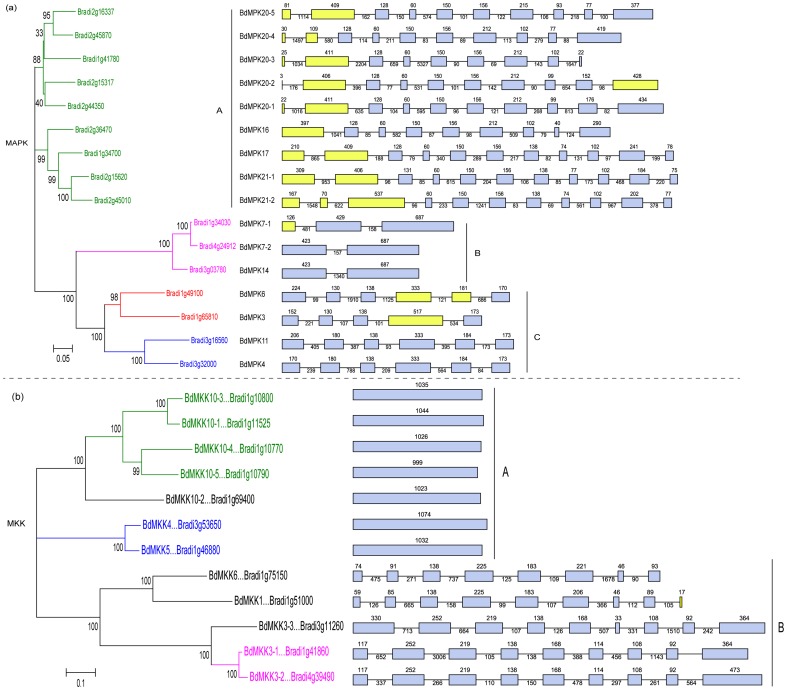
Phylogenetic analysis (Left) and exon-intron structures (Right) of *B. distachyon* MAPKs (a) and MAPKKs (b). The gene names of each gene cluster or supercluster originated from tandem or segmental duplication event were indicated with the same color in the phylogenetic tree except the black color. Only coding exons, represented by blue or yellow boxes, were drawn to scale. The black solid lines connecting two exons represented introns. Exons with different structures among the two family genes in the same subgroups were marked in yellow. Numbers above the exons or below the introns of each gene structure represented the size of exon and intron, respectively.

To obtain some insight into the gene structures of the MAPK and MAPKK family genes, their exon/intron organizations were analyzed. It was found that all the members of MAPK family had introns varying from three to eleven, while 58.3% of the total MAPKK genes had no intron. The MAPK and MAPKK genes could be divided into three and two subgroups based on their exon/intron structures, respectively (Figure 6). The gene structures of MAPK family were more divergent than that of MAPKK family. Some losses or gains of exons were also identified during the evolution of these two family genes at the 5′ end or 3′ terminal, and even in the middle of their sequences such as *BdMPK7-1, BdMKK1* and *BdMPK6*. Nonetheless, the exon/intron structures of each gene cluster originated from tandem or segmental duplication event in the two gene families tended to share similar structure organizations except tiny difference. One such example was *BdMPK4* and *BdMPK11*. These findings suggested these two family genes preserved a relatively constant exon-intron composition in each subgroup of them during the evolution of the *B. distachyon* genome. Moreover, we examined the *B. distachyon* MAPKs and MAPKKs for the occurrence of alternative mRNA splicing. The cDNA sequences obtained in our study were compared with all available full-length cDNA and EST sequence data. The investigations identified two alternatively spliced mRNAs for each *BdMKK1* and *BdMKK6*, respectively. The differential splicing events occurring in the two genes resulted in the difference in the C-terminal part of their translated protein (Figure S2). And the differentially spliced transcript forms (BdMKK6.1 and BdMKK6.2) of *BdMKK6* were exemplarily selected for the following interaction and expression analysis in order to assess whether they have similar function.

In addition, we also analyzed the promoter sequences in 1 kb region upstream of the predicted MAPK and MAPKK encoding genes as described in *M. truncatula* [52]. Four regulatory elements including the WBOX cassettes, CBF boxes, DRE boxes and the GCC motif implicated in either response to pathogens or plant stress were identified as being overrepresented in the 1 kb region upstream of the two gene families. The analysis results showed that WBOX element existed in all the members of the two families, averaging 7.38 per MAPK gene and 8.15 per MAPKK gene, respectively (Table S2). And 62.5% of MAPK genes and 75% MAPKK genes contained at least 6 predicted WBOXs. In contrast, the average numbers of other element types were 1.75 (CBF), 2.06 (GCC) and 1.06 (DRE) for MAPK family and 0.92 (CBF), 2.25 (GCC) and 0.5 (DRE) for MAPKK family, respectively. Of the predicted MAPK genes, 50% contained the four multiple boxes, while there was only 33.3% of MAPKK genes including these four multiple boxes. Moreover, we see no clear evidence of a correlation between the arrangement of these promoter cassettes (WBOX, CBF, DRE and GCC) and *in silico* expression via EST counts (Table S2).

### Synteny analyses of MAPKs and MAPKKs between *B. distachyon* and other two plant species of rice and *Arabidopsis*

To further explore the origin and evolutionary process of *B. distachyon* MAPK and MAPKK genes, we analyzed the comparative synteny map among *B. distachyon*, rice and *Arabidopsis* genomes. *Arabidopsis* and rice are two most important eudicot and monocot model plant species and the functions of many *Arabidopsis* and rice genes have been well characterized. Genomic comparison is a quick way to transfer genomic knowledge acquired in one taxon to a less-studied taxon [44], [53]. Thus, through comparative genomics analysis we could confidently infer the functions of *B. distachyon* MAPK and MAPKK genes based on their *Arabidopsis* and rice homologs.

**Figure 7:**
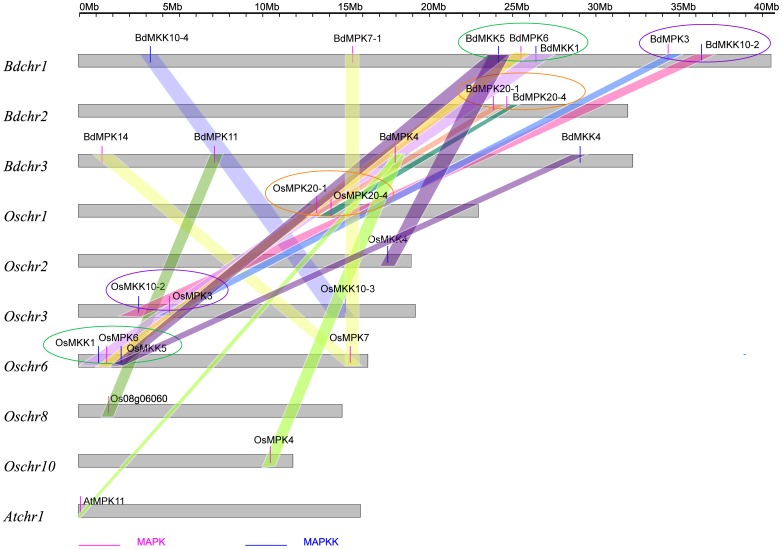
Syntenic analyses of MAPKs and MAPKKs between *B. distachyon* and the other two plant species of rice and *Arabidopsis*. To identify the species of origin for each chromosome, a species acronym is included before the chromosome name: At, *Arabidopsis thaliana*; Os, *Oryza sativa*; Bd, *B.distachyon*. Different color bars connect syntenic regions between *B. distachyon* and the other two plant species of *Arabidopsis* and rice chromosomes. The region of *B. distachyon* tightly linked cluster likely to be syntenic with that of the rice tightly linked cluster by translocation, insertion or other events was indicated with the ellipse in the same color.

Through large-scale synteny analysis between *B. distachyon* and the other two species of rice and *Arabidopsis*, 50% of MAPK genes and 42% of MAPKK genes from *B. distachyon* were identified to be synteny with their orthologs of rice genome, while there was only one gene (*BdMPK4*) in these two families found being synteny with *AtMPK11* of *Arabidopsis* (Figure 7). The result was consistent with the current understanding of plant evolutionary history. From Figure 7, we also found that their homologs of the tightly linked genes in the *B. distachyon* chromosome were still tightly linked in the rice chromosome with identical or opposite orientation such as the pair of *BdMPK20-1/BdMPK20-4* and *OsMPK20-1/OsMPK20-4*, the pair of *BdMKK5/BdMPK6/BdMKK1* and *OsMKK1/OsMPK6/OsMKK5*, and the pair of *BdMPK3/BdMKK10-2* and *OsMKK10-2/OsMPK3*. Such instance could be explained as random translocations and insertion events or thought of that the progenitor species had donor region (a tightly linked cluster of related or unrelated sequences in one part of the genome) and the *B. distachyon* or rice accepted this region in the process of species evolution. More challenging for syntenic interpretation were cases where duplicated *B. distachyon* MAPK or MAPKK genes yet corresponded to one or two rice homologs and the closest homologs in the phylogenetic tree were not the same with that of syntenic analysis such as *BdMPK7-1/BdMPK14-OsMPK14* and *BdMKK4/BdMKK5-OsMKK4/OsMKK5* (Figure 7). Of course, there were also some MAPK and MAPKK genes of *B. distachyon* that could not mapped to any syntenic blocks with the other two species. However, we could not conclude that the two families from *B. distachyon*, rice and *Arabidopsis* did not share a common ancestor. That may be the reason that their genomes have undergone multiple rounds of significant chromosomal rearrangement and fusions, followed by selective gene loss, which can severely obscure the identification of chromosomal syntenic blocks during the speciation of *B. distachyon*, rice and *Arabidopsis* [44].

### Analyses of the interactions between MAPK and MAPKK family members

**Figure 8:**
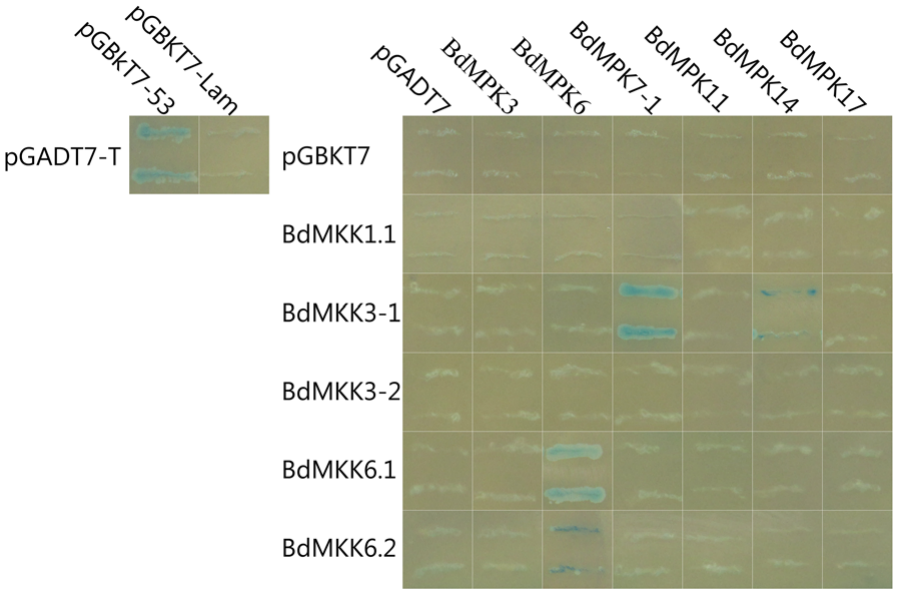
Comparative yeast two-hybrid interaction analyses of six BdMPKs with five BdMKKs. The yeast strain YH2Gold containing the indicated plasmid combinations was grown on nutritional selective medium minus His, Leu, Trp and plus X-α-Gal (SD/-His-Leu-Trp/X-α-Gal, TDO/X). Positive or negative control on the left corner showed the interaction between SV40 large T-antigen and murine p53 or SV40 large T-antigen and human lamin C (Lam) examined on TDO/X, respectively.

MAPK cascades are conserved signaling modules found in all eukaryotic cells, including plants, fungi and animals and they play a remarkably important role in plant signaling of a variety of abiotic and biotic stresses. However, So far only a few *Arabidopsis* MAPK cascade components and their interaction have been studied in detail. In order to unravel the interactions between the last two kinases within the MAPK cascade in *B. distachyon*, we studied their interactions between several genes of the two families using yeast two-hybrid assay. Four MAPKK genes (five proteins generated from alternative splicing) and six MAPK genes with more EST counts support were cloned into the respective DNA-binding domain and GAL4 activation domain plasmids. After transformation into the yeast strain YH2Gold, interaction was monitored by growth on interaction-selecting media lacking His. As shown in Figure 8, *BdMPKK3-1* exhibited a significant interaction with *BdMPK7-1* and weak interaction with BdMPK14, while its sister gene *BdMPKK3-2* derived from gene duplicate event did not show any affinity toward the pair of duplicated *BdMPK7-1/BdMPK14* and other MAPKK genes. The interactive partners between *BdMPKK3-1* and *BdMPK7-1/BdMPK14* were also found in their homologs of *Arabidopsis* which were activated by Pathogen and JA [5]. In addition, the two isoforms (BdMAKK6.1 and BdMAKK6.2) of *BdMKK6* generated by alternative splicing were also cloned into the DNA-binding domain plasmids in order to investigate whether they had similar function. From Figure 8, we can found that they indeed interacted with the same gene *BdMPK6* but had different affinity, which may be due to tiny difference of their amino sequences. Therefore, the exact structural features determining the specificity of complex formation remain to be uncovered. Similarly, the two isoforms also interacted only with *BdMPK6* and did not interact with the sister gene *BdMPK3* of *BdMPK6* as the case of *BdMPKK3-1*. So far, the interactive partner of *BdMKK6/BdMPK6* has not been found in their homologs of *Arabidopsis* and other plants. That is maybe the reason that there were few reports on the MAPKK family, especially on the MKK6 gene. More interestingly, the homologs of *BdMPK3/BdMPK6* in *Arabidopsis* are closely related genes that show a high level of functional redundancy [4]–[5] and always accompany together in the previous reported signaling pathway. However, the two duplicated genes of *BdMPK3/BdMPK6* in *B. distachyon* were not the case from the results of interactive yeast two-hybrid interaction assay, which indicted that sequence similarity and evolutionary history were not sufficient to predict MAPKK-MAPK interactions; their interaction profiles found in one model plant did not always exist in other model plants and also suggested that even a high conservation of certain MAPKKs or MAPKs does not necessarily represent the functional redundancy.

### Expression profiles of MAPK and MAPKK genes in *B. distachyon*, *Arabidopsis* and rice under normal grow and various abiotic stress conditions

#### The expression profiles of *B. distachyon* MAPK and MAPKK genes

**Figure 9:**
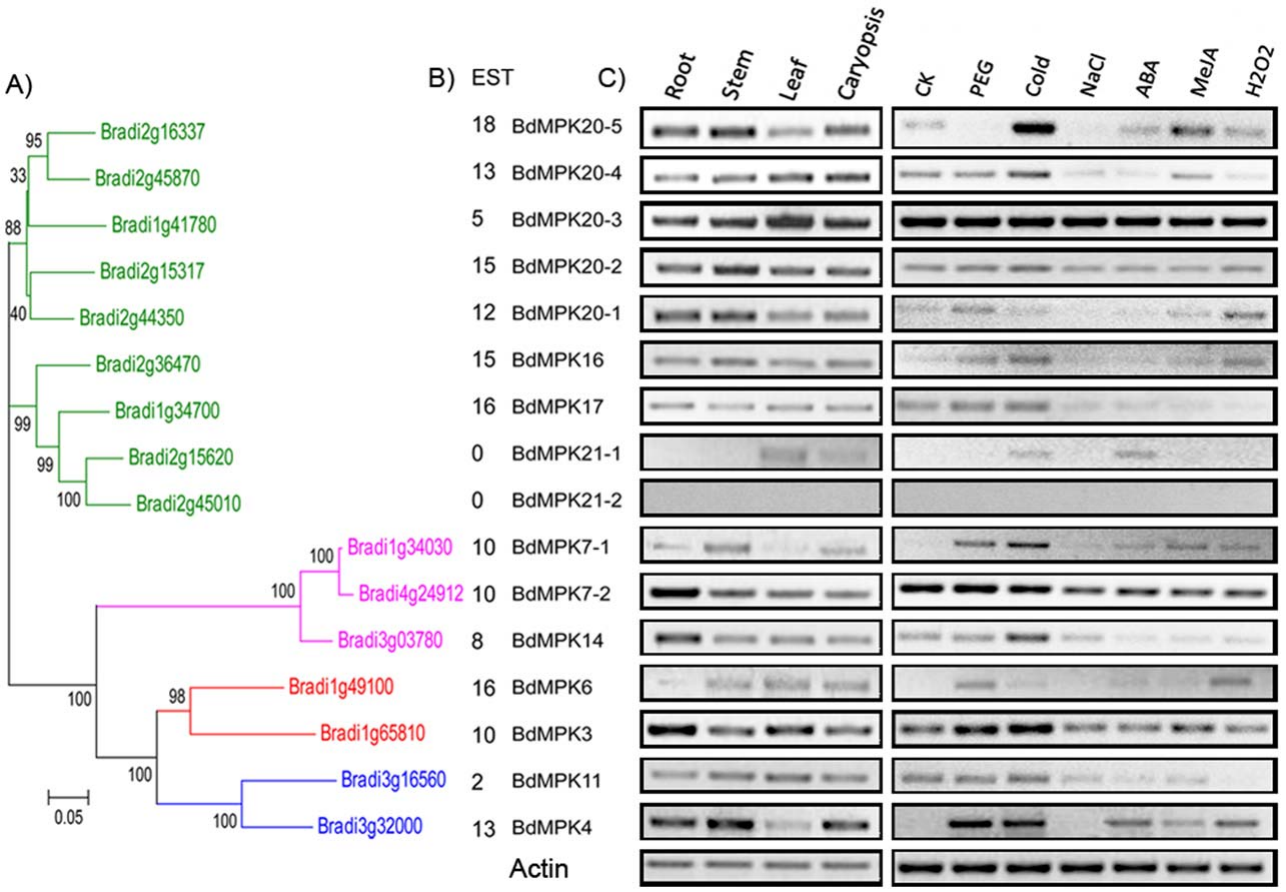
Phylogenetic (A), EST (B) and RT-PCR (C) analyses of *Brachypodium* MAPK genes. The gene names of each gene cluster originated from tandem or segmental duplication event were indicated with the same color.

To observe the organ-specificity of the two family members, we confirmed their transcript level in four different organs: roots, stems, leaves and caryopses. For MAPK family, the vast majority of its members were expressed in all the detected organs except the *BdMPK21-1* and *BdMPK21-2* (Figure 9). *BdMPK20-3* had higher expression level than other members of MAPK family in all the tested corresponding organs. The expression of *BdMPK6* was induced in stem, leaf and caryopses, while its closely duplicated sister gene *BdMPK3* was not the case. Similar result was observed in the duplicated pair of *BdMPK7-1/BdMPK7-2* and other duplicated gene pairs which both existed in MPAK and MAPKK families. These results showed that although the duplicated genes had higher similarities in amino acid, we can't speculate that they should have similar function or were involved the same signaling pathway. Therefore their expression intensity and/or organ-specificity varied depending on their specific function but not on the sequence similarity. In addition, we increased the mRNA amount of each organ and the cycles of RT-PCR in order to investigate whether *BdMPK21-1* and *BdMPK21-2* that were not detected in the same experimental condition as other members had trace expression in the undetected organs. However, we still could not detect the expression of *BdMPK21-1* in roots and stems or the expression of *BdMPK21-2* in all the four organs.

Compared with MAPK family, the expression intensity of each MAPKK member varied slightly greater in different organs than that of each MAPK member. For example, 81.25% of MAPK members had relatively uniform high level expression under our four detected tissues, while there were only 33.33% of MAPKK genes with similar expression level. Fifty percent of MAPKK genes were induced or up-regulated in caryopses compared with that in roots, whereas only 18.75% MAPK genes were the case and most of them had no significant difference. From these results, it is interesting to speculate that most of MAPK genes with relative uniform high expression levels in all the *B. distachyon* detected organs might play key roles in plant development and half of MAPKK genes induced in caryopses may represent their unique functions in this developmental stage. However, more researches are needed to determine the functions of the two family genes through other biological experimental technology.

**Figure 10:**
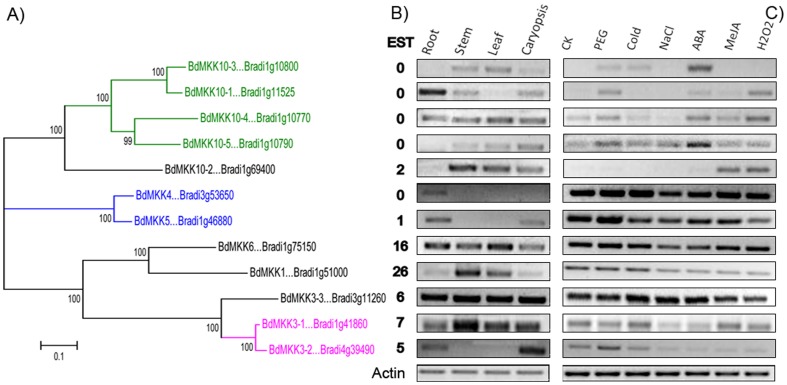
Phylogenetic (A), EST (B) and RT-PCR (C) analyses of MAPKK genes. The gene names of each gene cluster originated from tandem or segmental duplication event were indicated with the same color (except the black color).

Evidence is accumulating that MAPK and MAPKK proteins are involved in responding to various abiotic stresses. Thus, we used RT-PCR method to examine the expression of the two family genes in response to six different abiotic stresses or hormone treatments: drought, salinity, cold, ABA, MeJA and H_2_O_2_. From the results we found that each member of the two family genes showed differential expressions in response to at least two treatments except for BdMPK21-2 that did not show any detectable expression in all treatments as that in the organs mentioned above, which indicated that either this gene is expressed in other organs or under other stress treatments, expressed with low level or it is a pseudogene (Figure 9, Figure 10). And 61% of the two family genes were induced or constitutively expressed in all the six treatments, implying their important roles in the stress tolerance. Of course, there were some genes that were up-regulated and down-regulated in one treatment but not in all the treatments. For example, 43.75% of MAPK family genes were up-regulated and only one gene was down-regulated compared with the control in the PEG treatment, while 66.7% of MAPKK genes were up-regulated and none of them were down-regulated. More than fifty six percent of MAPK family genes were up-regulated and none of them was down-regulated compared with the control in the cold treatment, while 16.7% of MAPKK genes were up-regulated and one of them was down-regulated. Taken together, the majority of the two family genes were expressed or induced under the stress conditions of drought, salt and cold, ABA, MeJA and H_2_O_2_ respectively, implying that these members might be putative regulators in response to various abiotic stress or hormone treatments. Moreover, we also found that the genes of the two families with EST support were expressed with higher level or in a much wider range of libraries, including those constructed from various stress conditions and organs than that with no EST support. However, there was no clear evidence of a correlation between the expression intensity by RT-PCR and the EST counts of each gene from the two families. For example, the four genes *BdMKK10-1, BdMKK10-3, BdMKK10-4* and *BdMKK10-4* derived from tandem duplication event had no EST support, but varying expression intensity was detected in some tissues or stress conditions, which showed that the expression patterns of the two family genes were highly variable and were not strongly associated with their EST counts or sequence similarity.

#### The expression profiles of *Arabidopsis* MAPK and MAPKK genes

**Figure 11:**
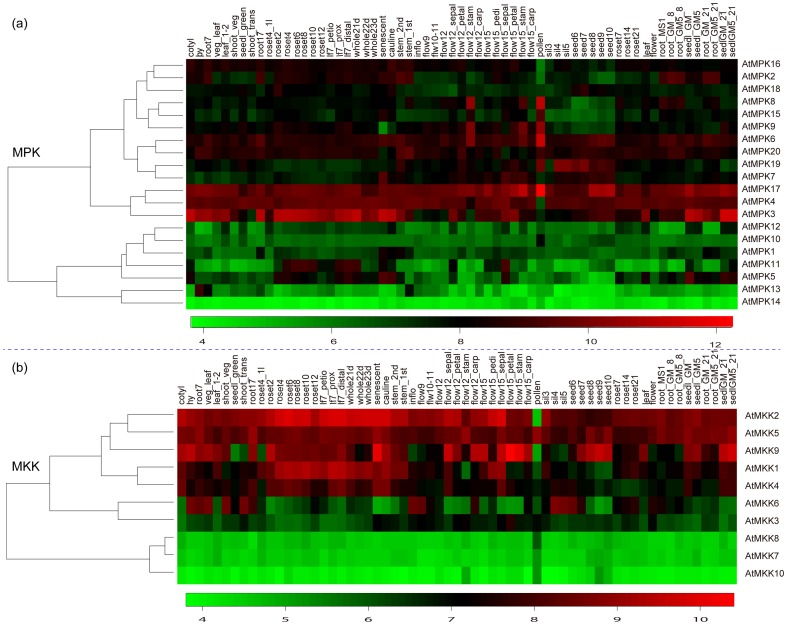
Expression profiles of 20 MAPK genes (a) and 10 MAPKK (b) genes in different *Arabidopsis* **tissues.** Details of the experimental conditions are provided in Table S3. Log2 based value was used to create the heatmap. Difference in gene expression changes is shown in color as the scale.

To observe the organ-specificity and the responses to stress conditions of the two family members in *Arabidopsis*, a total of 4 experiments including 201 hybridizations from the Affymetrix *Arabidopsis* genome array were obtained. All the genes of the two families corresponding to 30 probe sets were identified in the 4 experiments. Detailed expression of the two family genes under different developmental stages was provided in Table S3. Heatmap representation of expression profiles of these two family genes during *Arabidopsis* development was shown in Figure 11. For the MAPK family genes, *AtMPK5* and *AtMPK11* had higher expression in rosette, flower, and seeds than that of other organs. *AtMPK1, AtMPK10, AtMPK12, AtMPK13* and *AtMPK14* were expressed with low abundance in nearly all the organs, whereas *AtMPK3, AtMPK4, AtMPK6, AtMPK17* and *AtMPK20* were constitutively expressed with high abundance in nearly all the organs (Figure 11a). More interestingly, this case was consistent with the expression profiles of their homologs in *B. distachyon*. For example, *BdMPK3, BdMPK4*, *BdMPK6, BdMPK17* and all the members of *BdMPK20* clade were constitutively expressed with high level in nearly all the *B. distachyon* detected organs. For the MAPKK family genes, *AtMKK2, AtMKK5* and *AtMKK9* clustered together in the heatmap with high expression in the majority of organs, while the clustered *AtMKK7, AtMKK8* and *AtMKK10* had low expression in nearly all the organs. The expression level of other members varied depending on different organs.

In addition, we also identified the expression profiles of the two family genes under cold, drought and salt stress conditions through analysis of publicly available microarray data sets. Details of the experimental analytic result were provided in Table S4. A total of 5 MAPK genes (*AtMPK3, AtMPK5, AtMPK7, AtMPK11 and AtMPK19*) were significantly up-regulated and 4 MAPK genes (*AtMPK7, AtMPK8, AtMPK16 and AtMPK20*) were significantly down-regulated under the cold or salt stress conditions according the strict criteria of up-regulated or down-regulated genes described in the method section. Among the 5 up-regulated genes, *AtMPK3* was identified under the cold treatment, which was consistent with previous report that the expression of *AtMEKK1* and *AtMPK3* could be induced by cold [54], Actually, *AtMPK3* was one of the best-characterized members in the *Arabidopsis* MAPKs and involved in many signal pathways activated by a diversity of stimuli including abiotic stresses, pathogens and oxidative stress. For the MAPKK family, four genes were up-regulated or down-regulated under one or two stress conditions and *AtMKK9* was up-regulated under both the cold and salt treatments. However, none of MAPK and MAPKK members was identified to be significantly up-regulated or down-regulated under the drought treatment. Besides these genes in the two families which showed significant changes in response to the stress conditions, there are also lots of genes showing tiny changes under different stress treatments as provided in Table S4.

#### The expression profiles of rice MAPK and MAPKK genes

**Figure 12:**
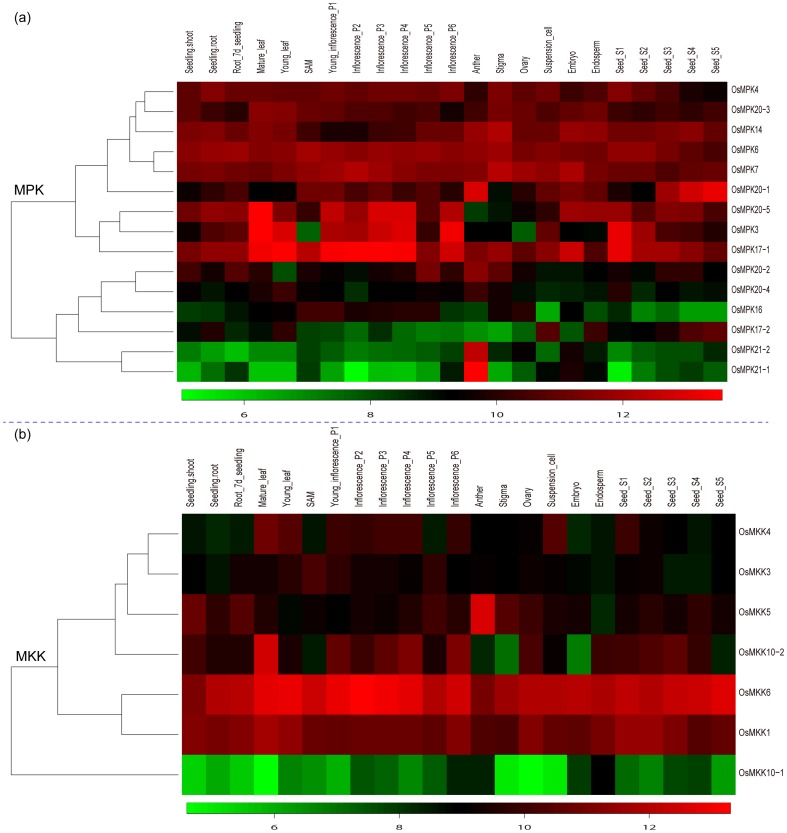
Expression profiles of 15 MAPK genes (a) and 8 MAPKK (b) genes in different rice tissues. Details of the experimental conditions are provided in Table S4. Log2 based value was used to create the heatmap. Difference in gene expression changes is shown in color as the scale.

Similarly, the expression profiles of the two family genes in rice under different developmental stages and the three stress conditions mentioned above were also examined as that in *Arabidopsis* using the available microarray database. A total of five experiments including 70 hybridizations from the Affymetrix rice genome array were obtained. Twenty two genes of the two families corresponding to 27 probe sets were identified in the five experiments and there was only one representative MAPKK genes whose corresponding probe was not found. Detailed expression profiles of the two family genes under different developmental stages were provided in Table S5. Heatmap representation of expression profiles of these two family genes during rice development was shown in Figure 12. There were almost 60% of MAPK genes clustered together in the heatmap with high expression in nearly all the organs except *OsMPK20-5* in anther and *OsMPK3* in ovary and shoot apical meristem (SAM). The expression profiles of these genes happened to be consistent with that of their homologs in *B. distachyon* and *Arabidopsis* except *AtMPK7* and *AtMPK14*, especially the *AtMPK14* which not only had low expression in all the detected organs but also had no significant fluorescent signal in all the stress conditions. More interestingly, the expression profiles of two paralogs *OsMPK17-1* and *OsMPK17-2* originated from tandem duplication event were quite different under the majority of rice developmental stages, which may reveal that after duplication one daughter gene retained the ancestral function while the other acquired new functions [55]. However, the expression profiles of duplicated paralogs *OsMPK21-1* and OsMPK21-2 showed highly similar expression intensities, which may indicate subfunctionalization in the course of evolution. Beside this case, there were also several cases existing in the homologs of MAPKK family. For example, *OsMKK1* and *OsMKK6* clustered together in the heatmap were constitutively expressed with high expression in all the tissues and their homologs in *B. distachyon* also had similar expression profiles. *OsMKK10-1* had low expression in all the tissues and its closest related gene *AtMKK10* in the phylogenetic tree showed similar expression profile.

When using microarray data to investigate the response of the two family genes to abiotic stresses, we found that 80% of OsMPK genes and 50% of OsMPKK genes were up-regulated or down-regulated under one or more stress conditions (Table S4). For example, *OsMPK17-1* and *OsMKK4* were both up-regulated under the three stress conditions, while the duplicated pair of *OsMPK20-2* and *OsMPK20-3* were both down-regulated. However, we also found that some closely related genes of the two families in the three species with similar expression profiles under different developmental stages did not show similar expression profiles when they were under the three stress conditions and *vice versa* (Table S4).

### Pairwise comparisons of the expression profiles of putative orthologous or paralogs pairs existing in the two family genes of *B. distachyon* and rice under different light and temperature conditions

**Figure 13:**
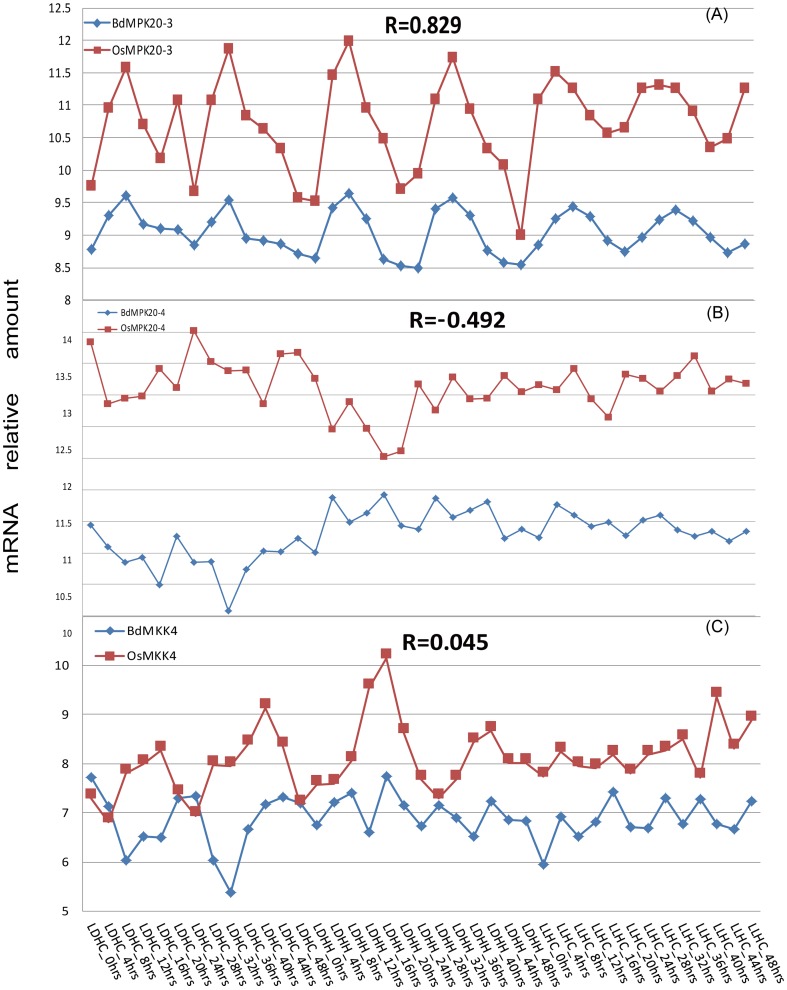
Pairwise comparisons of the expression profiles of putative orthologs between rice and *B. distachyon* **MAPKs and MAPKKs.** The mRNA relative amount represents the log 2 based value of expression intensity and the expression profiles of the two family genes in rice and *B. distachyon* were provided in Table S6. The treatment time (h) under the particular light and temperature condition is presented on the x-axis. R indicates the correlation coefficient of the expression profiles between orthologs or paralogs pairs under the corresponding light and temperature treatments. The positive (A), negative (B) and no obvious correlation (C) were detected from our identified 17 pairs of putative orthologs or paralogs in rice and *B. distachyon* MAPKs and MAPKKs.

The expression profiles of the two family genes of *B. distachyon* and rice under different light and temperature conditions were shown in Figure S3 and Figure S4. Most of them had comparative uniform expression profiles, while the expression intensity of *BdMPK14, BdMPK20-3, BdMPK3-3, BdMKK4* and *OsMPK20-1, OsMPK20-2, OsMPK20-5, OsMKK4* varied comparatively greater than that of others. In order to investigate whether the expression profiles of these duplicated orthologs or paralogs pairs at the terminal nodes of phylogenetic tree were correlated in the two plant species, we compared their expression profiles of them under different light and temperature conditions (Table 2). The results showed that there were only three sets of data whose correlation coefficients were greater than 0.5, indicating a positive correlation between these orthologs pairs under different light conditions such as the pair of *BdMPK20-3* and *OsMPK20-3* (Figure 13A), while there was a set of data, whose correlation coefficients were not lower than -0.5 but may have certain negative correlation such as the pair of *BdMPK20-4* and *OsMPK20-4* (Figure 13B). In addition, we also found that some sets of data whose correlation coefficients were very small and had no clear positive or negative correlation under the different light and temperature conditions such as the pair of *BdMKK4* and *OsMKK4* (Figure 13C). The comparison analysis results indicated that the duplicated gene pair may remain parts of its ancestral function or showed divergent function under different stress conditions in the evolutionary process. However, the reasons for expression divergence of the duplicated genes in many gene families remain unclear. Previous study showed that the long-term evolutionary fate of duplicated orthologs or paralogs pairs would be determined by the functions of the duplicated genes. There are four types of functional differentiation following gene duplication: pseudogenization, conservation of gene function, subfunctionalization and neofunctionalization [55]. Many duplicated genes may be lost from the genome after the duplication events, and neofunctionalization and subfunctionalization are the major factors for the retention of new genes. Our analytic results of the duplicated gene pairs were consistent with this viewpoint. Besides these, the expression patterns of the two isoforms (BdMKK6.1 and BdMKK6.2) derived from alternative splicing event were also analyzed. More interestingly, the expression of one isoform BdMKK6.1 with longer protein sequence was identified in all the detected tissues and stress conditions and another one BdMKK6.2 was just opposite by traditional RT-PCR. When using the microarray data under different light conditions, we found that the isoform BdMKK6.1 happened to be expressed with higher level than BdMKK6.2. However, the correlation coefficient of their expression profiles was very low (0.181). It may be the reason that they were not involved in this light pathway but played important roles in other signal pathways or that they indeed had some difference in their functions.

**Table 2:** Pearson correlation coefficients for the expression profiles of orthologs or paralogs pairs.

Family	Gene	Gene	Similarity^a^	Correlation coefficient
**MAPK**	*BdMPK3*	*OsMPK3*	91.9%	−0.113
	*BdMPK4*	*OsMPK4*	94.4%	0.113
	*BdMPK6*	*OsMPK6*	94.5%	0.594
	*BdMPK14*	*OsMPK14*	96.2%	0.311
	*BdMPK16*	*OsMPK16*	81.3%	−0.345
	*BdMPK17*	*OsMPK17-1*	86.5%	−0.097
	*BdMPK20-1*	*OsMPK20-1*	90.4%	0.276
	*BdMPK20-3*	*OsMPK20-3*	65.6%	0.829
	*BdMPK20-4*	*OsMPK20-4*	86.8%	−0.492
	*BdMPK21-1*	*OsMPK21-1*	81.9%	0.075
**MAPKK**	*BdMKK1*	*OsMKK1*	87.2%	−0.289
	*BdMKK3-1*	*BdMKK3-2*	90.2%	0.263
	*BdMKK4*	*OsMKK4*	90.8%	0.045
	*BdMKK5*	*OsMKK5*	82.9%	0.396
	*BdMKK6*	*OsMKK6*	93.3%	0.204
	*BdMKK10-2*	*OsMKK10-2*	84.8%	−0.066
	*BdMKK10-4*	*BdMKK10-5*	69.9%	0.657

a represents the similarity of amino acid.

### Conclusion

Out of many signaling pathways involved in biotic and abiotic stress response in plants, MAPK cascade is one of the major pathways. This signaling module links external stimuli with several cellular responses and is evolutionary conserved among eukaryotic organisms [2], [56]. In plants, MAP kinases are represented by multigene families and are involved in efficient transmission of specific stimuli and also involved in the regulation of the antioxidant defense system in response to stress signaling [54]. So far, significant progress has been made toward the identification and characterization of MAPK and MAPKK gene families in several model plants, while no systematic analysis for the two family genes was reported in *B. distachyon*. In this present study, we carried out a genome-wide survey of the two gene families in *B. distachyon* and characterized them on the bases of structural diversity, phylogenetic relationship, conserved protein motif, chromosomal location, gene duplication, exon/intron organization, promoter region, interaction between them and expression profiles. In addition, we also analyzed the expression patterns of the two family genes in *Arabidopsis* and rice under different developmental stages as well as under various stress conditions and comparatively analyzed the expression profiles of putative orthologous or paralogs pairs existing in the two family genes of *B. distachyon* and rice under different light and temperature conditions. Therefore, our genomic and bioinformatics analysis of the two family genes and proteins presented in this work would provide an important foundation for the further functional dissection of the last two kinases of MAPK cascade involving in the important signaling pathways in different organs and under different stress conditions. Beside these, overexpression, knockdown or mutagenesis, and promoter analysis of selected members of the two families are underway in our laboratory so that we can accurately determine molecular pathways in the MAPK and MAPKK gene families.

## Supporting Information

Figure S1 **A detailed motif and subdomain introductions for** ***Brachypodium*** **MAPKs.**
(TIF)

Figure S2 **Schematic depictions of alternatively spliced BdMKK1 and BdMKK6 genes.**
(TIF)

Figure S3 **Expression profiles of BdMPKs (a) and BdMKKs (b) under different light and temperature conditions.** Details of the experimental conditions are provided in Table S6. Log2 based value was used to create the heatmap. Difference in gene expression changes is shown in color as the scale.
(TIF)

Figure S4 **Expression profiles of OsMPKs (a) and OsMKKs (b) under different light and temperature conditions.** Details of the experimental conditions are provided in Table S6. Log2 based value was used to create the heatmap. Difference in gene expression changes is shown in color as the scale.
(TIF)

Table S1 **The primer sequences used for RT-PCR amplification and gene cloning of the two family genes.**
(XLSX)

Table S2 **Annotation of the** ***Brachypodium distachyon*** **MAPK and MAPKK gene families.**
(XLSX)

Table S3 **The expression profiles (log2 based value) of the two family genes of** ***Arabidopsis*** **under different developmental stages.**
(XLSX)

Table S4 **The expression profiles of the two family genes of** ***Arabidopsis*** **,** ***Brachypodium*** **and rice under three different abiotic stress conditions.**
(XLSX)

Table S5 **The expression profiles (log2 based value) of the two family genes of rice under different developmental stages.**
(XLSX)

Table S6 **The expression profiles (log2 based value) of the two family genes of** ***Brachypodium*** **and rice under different and temperature conditions.**
(XLSX)
