# Supplementary figures and images for: Genome-Wide Identification and Analysis of MAPK and MAPKK Gene Families in Brachypodium distachyon
Source: PLoS One. 2012 Oct 17;7(10):e46744. doi: 10.1371/journal.pone.0046744 (PMC3474763; doi:10.1371/journal.pone.0046744)

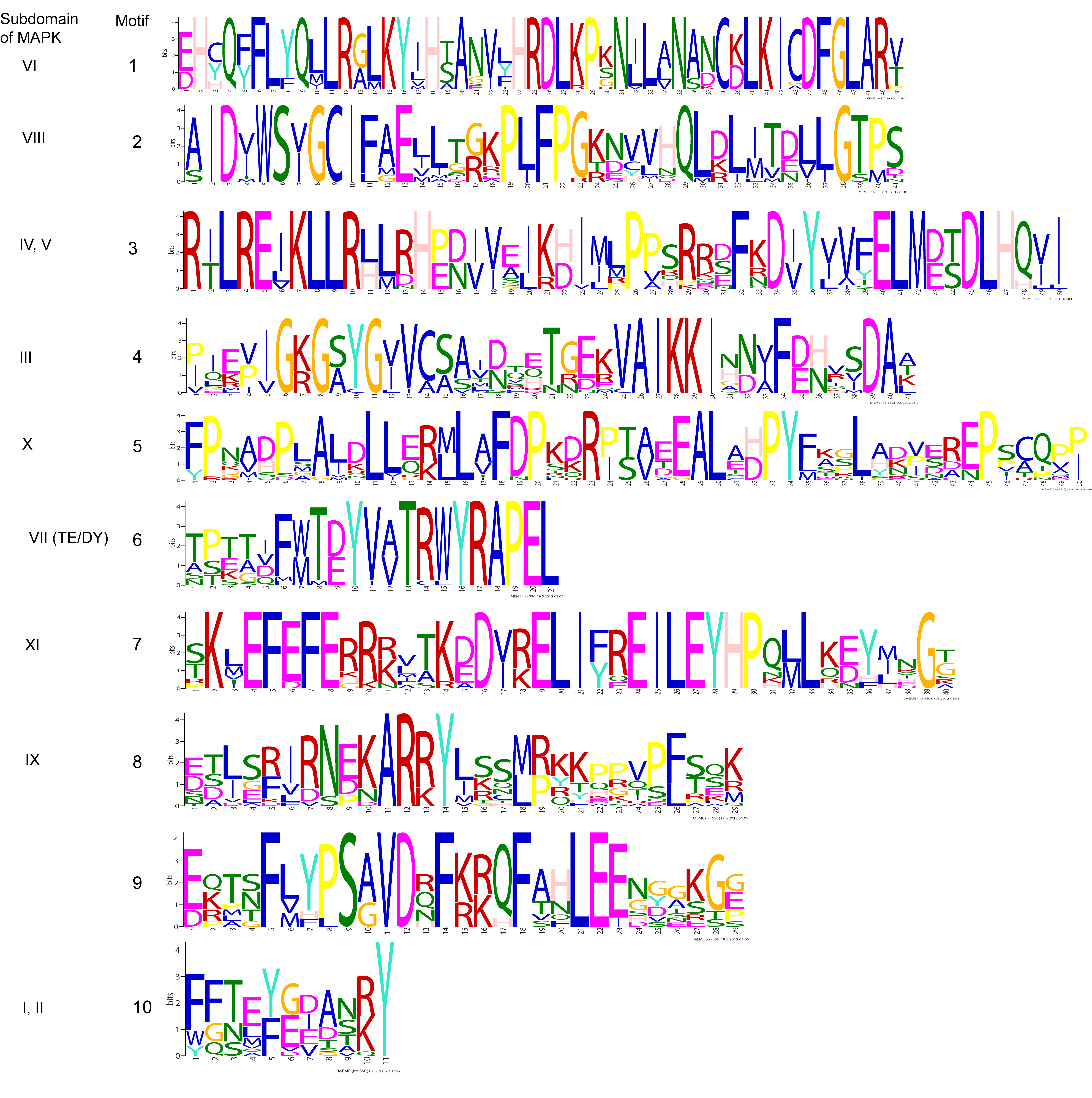

Supplement: Figure S1 — A detailed motif and subdomain introductions for Brachypodium MAPKs. (TIF) [file pone.0046744.s001.tif]

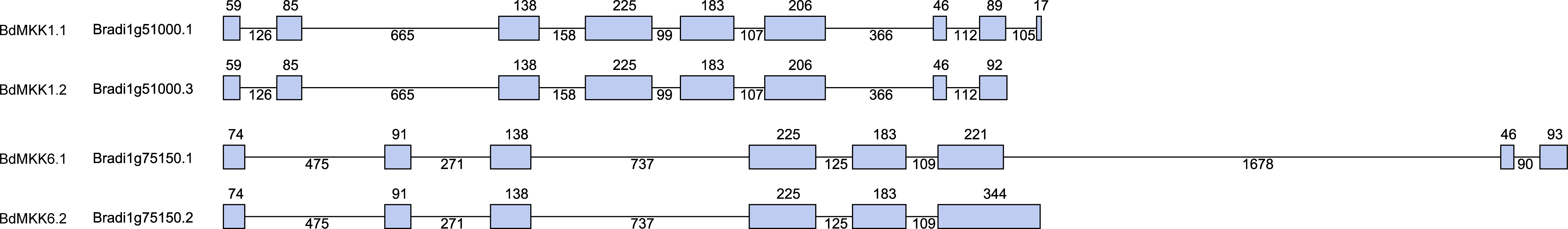

Supplement: Figure S2 — Schematic depictions of alternatively spliced BdMKK1 and BdMKK6 genes. (TIF) [file pone.0046744.s002.tif]

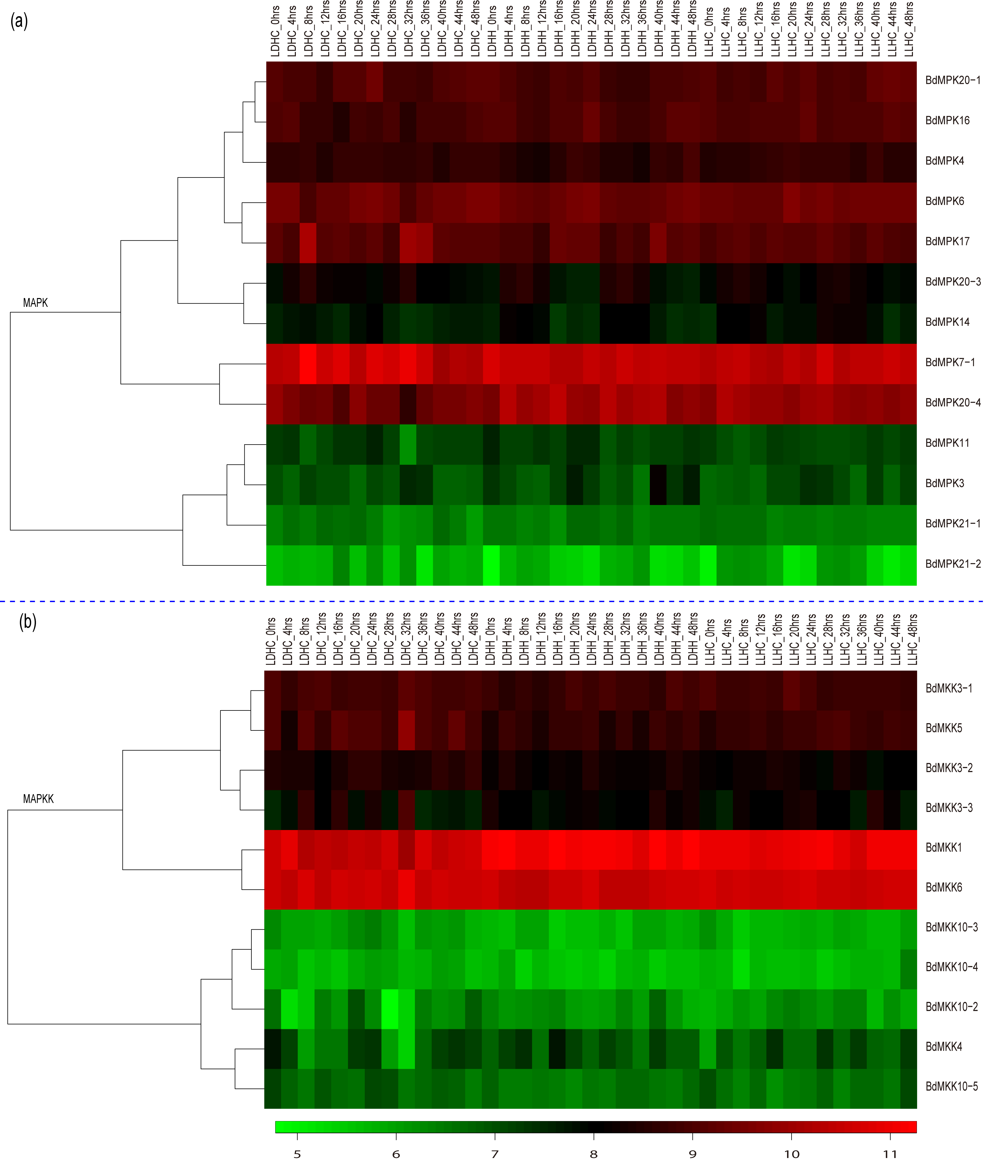

Supplement: Figure S3 — Expression profiles of BdMPKs (a) and BdMKKs (b) under different light and temperature conditions. Details of the experimental conditions are provided in Table S6. Log2 based value was used to create the heatmap. Difference in gene expression changes is shown in color as the scale. (TIF) [file pone.0046744.s003.tif]

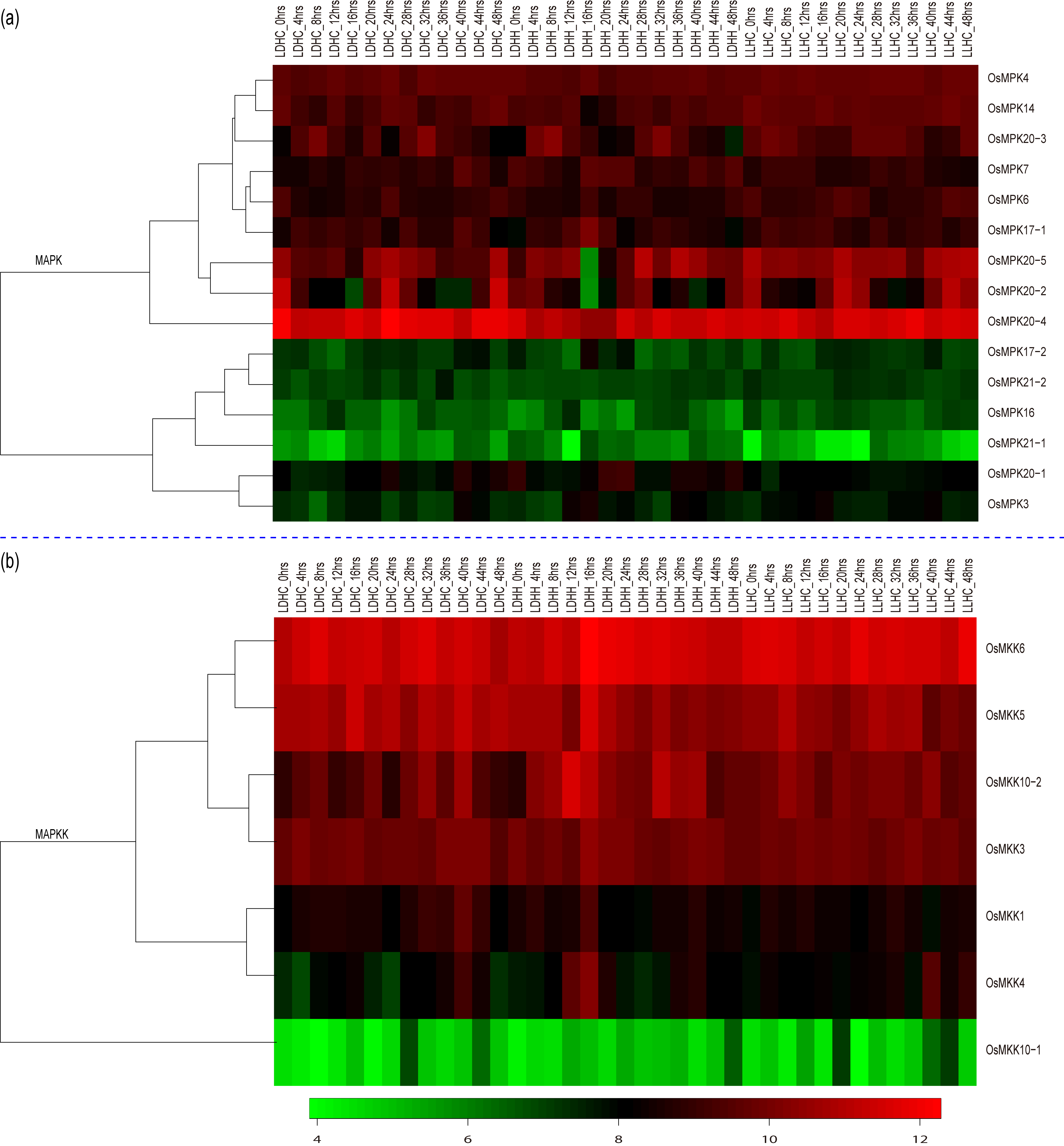

Supplement: Figure S4 — Expression profiles of OsMPKs (a) and OsMKKs (b) under different light and temperature conditions. Details of the experimental conditions are provided in Table S6. Log2 based value was used to create the heatmap. Difference in gene expression changes is shown in color as the scale. (TIF) [file pone.0046744.s004.tif]
